# Pregnancy from a Legal Standpoint—III

**Published:** 1909-03-20

**Authors:** 


### Medico-Legal Points.

### PREGNANCY FROM A LEGAL STANDPOINT—III.

### Infanticide.

In general, it is the mother of the child who is charged with the murder, and in this case it may be necessary, in order to connect her with the child, to determine whether she has or has not been recently delivered. Medical evidence may show that the date of delivery does or does not correspond with the date of the birth and death of the child. The usual appearances in cases of recent delivery, both in the living and dead body, necessarily vary according to the time at which the examination is made. Toulmouche has reported in detail several cases showing the post-mortem appearances met with at different dates.

Infanticide is the killing of a child immediately after it is born. The felonious destruction of the fœtus *in utero* is more properly called fœticide, or criminal abortion. In every case in which an infant is found dead, and its death becomes the subject of judicial investigation, the great questions which present themselves for inquiry are:—(1) What is the age of the child? (2) Was the child born alive? (3) If born alive, how long had it lived? (4) If born alive, by what means did it die? If it be proved that its death was owing to violence, it is then to be ascertained who the murderer of it is. If suspicion fall upon the mother, it is to be determined—(1) Whether she has been delivered of a child? and (2) whether the signs of a delivery correspond as to time, etc., with the appearances developed in the child? There are two ways in which a child may be born alive—(1) the cord may be pulsating, showing that it is alive, yet it may not respire; (2) it may be born and respire.

### What is Live Birth?

To constitute live birth, the child must be living after the whole body has been brought into the world, and it must have an independent circulation; but this does not imply the severance of the umbilical cord. The expert medical evidence is all important in cases of infanticide, because it is generally by that evidence alone that it can be shown that the child was born alive—an essential point, difficult often to prove, and one which never arises in other kinds of murder.

The law will not assume that the child was born alive; the burden of proof of this is thrown on the prosecution. If the child is killed when half expelled from the maternal parts, it is not born alive, and no crime known to our law is committed. To justify a conviction on an indictment charging a woman with the wilful murder of a child of which she was delivered, and which was born alive, the jury must be satisfied affirmatively that the whole body was brought alive into the world; and it is not sufficient that the child has breathed in the progress of the birth. Mr. Justice Littledale said:—"With respect to the birth, the being born must mean that the whole body is brought into the world, and it is not sufficient that the child respires in the progress of the birth. Whether the child was born alive or not depends mainly upon the evidence of the medical men. None of them say that the child was born alive, they only say that it had breathed, and if there is all this uncertainty among these medical men, perhaps you would think it too much for you to say that you are satisfied that the child was born alive."

### Judicial Rulings.

So where, upon an indictment containing a count for murder by stabbing, and a count charging that before the child was completely born the prisoner stabbed it with a fork, and that it was born, and then died of the stab, it was proved that a puncture was found on the child's skull, but when that injury was inflicted did not appear, and some questions were asked as to whether the child had breathed. Parke, J., said: "The child might breathe before it was born, but its having breathed is not sufficiently life to make the killing of the child murder; there must have been an independent circulation in the child, or the child cannot be considered as alive for this purpose."

Where one count charged that the prisoner, being big with a female child, "did bring forth the same alive," and then in the usual manner alleged the murder of the child by choking it with a handkerchief; and there was strong evidence to prove that the child had been wholly produced alive from the prisoner's body, and that she had strangled it; but it was also clearly proved by the surgeon who examined the body of the child that it must have been strangled before it had been separated from the mother by the severance of the umbilical cord, and the surgeon further stated that a child has, after breathing fully, an independent circulation of its own, even while still attached to the mother by the umbilical cord, and that in his judgment the child in question had breathed fully after it had been wholly produced and had therefore an independent circulation of its own before and at the time it was strangled, and was then in a state to carry on a separate existence. Erskine, J., directed the jury that if they were satisfied that the child had been wholly produced from the body of the prisoner alive, and that the prisoner wilfully strangled the child after it had been so produced, and while it was alive, and while it had, according to the evidence of the surgeon, an independent circulation of its own, he was of opinion that the charge was made out, although the child, at the time it was so strangled, still remained attached to the mother by the navel-string. The jury found the prisoner guilty, and upon a case reserved the judges held the conviction right. But if a child be actually wholly produced alive it is not necessary that it should have breathed to make it the subject of murder.

In the great majority of cases the crime is committed at birth or directly afterwards. It is immaterial, however, whether the age of the child is measured by minutes or days, since "it is equally murder to destroy the life of a child which has been completely born as it would be feloniously to kill an adult." Nor is it necessary that the child shall have reached the full term of intra-uterine development, provided it has lived after its birth.

### Pulmonary Evidence of Live Birth.

Was the child born alive? The difficulty which so frequently exists in giving a confident answer to this question arises from the absence of any sign by which to distinguish death just before birth (in the strict legal sense) from death just after birth. The medical witness must base his conclusions chiefly on the evidence supplied by the lungs. If the child has never breathed at all, the lungs will show the fact beyond all reasonable doubt, and, in the absence of evidence to the contrary, there is the strongest presumption of still-birth. But the converse is not true. Proof that the child has breathed is not proof that it was born alive, because it is fully established that a child can breathe and cry as soon as its head is outside the vaginal passage, and therefore before it is legally born, although such breathing does not produce the full effects normally resulting from respiration freely performed after complete expulsion. In the latter case well-marked changes occur in the volume, the colour, the condition of the air-cells, the consistency and appearance and the specific gravity of the lungs in consequence of respiration. These changes require some little time to develop fully, and therefore, where respiration has been carried on for a very short time, or very feebly, the degree to which these lung changes have developed is a rough guide to the degree to which the function of respiration has been carried on.

The subject of child-murder has greatly attracted the attention of medical jurists by reason of the facility with which the crime may be perpetrated, and the great difficulty of bringing it home to the offender. There is usually considerable reluctance on the part of a coroner's jury to return a verdict of wilful murder, when the mother may be sent to take her trial at the assizes for murder. Usually when the evidence of guilt has been so clear that coroners' juries have found verdicts of wilful murder, the prisoners have been subsequently acquitted on their trials. From the nature of the medical proofs required, a conviction for child murder in England in the present state of the law seldom takes place. Notwithstanding the frequency of this crime, juries appear to shrink from returning a verdict of murder, even where the medical facts would fully justify it, but they almost invariably fall back upon the minor offence of which the accused person may be convicted, namely, that of concealment of birth. In some cases, however, under the direction of some of our judges, verdicts of manslaughter have been obtained.

In cases of infanticide there is a most difficult duty cast upon a medical witness. In the greater number of cases the woman is delivered in secrecy, and no one is present to give evidence respecting the birth of the child.

### The Medical Witness and Privilege.

Under these circumstances medical evidence is especially required. Here a medical man should be especially cautious in putting questions to a woman charged with this crime. As there is no special privilege granted to members of the medical profession, a witness must remember that there are no medical secrets. In a case in which a female was indicted for the murder of her infant a surgeon was called to prove certain confessions made to him by the woman during his attendance. He objected on the ground that he was then attending her as a private patient. Parke, J., said this was not a sufficient reason to prevent a disclosure for the purposes of justice, and he was ordered to answer the questions. ("Beck's Med. Juris.") At the meeting of the British Medical Association at Leeds, August, 1869, Bateman said, "There are many cases in which a doctor cannot discharge his duty to a sick person without putting questions, the replies to which may criminate the patient, or seriously affect his interest, and these replies the doctor is now called upon to communicate either in a civil or criminal court."

A case was mentioned in which two sisters were servants to an old lady. One of them became pregnant, miscarried, and was attended by a surgeon. The mistress, who knew all about the matter, retained the girl in her service, and left her a legacy at her death. The will was disputed by the heir-at-law on the ground of undue influence, and at the trial, in order to injure the girl's character, the surgeon was called, and asked for what illness he had attended her some years before. Believing that he had a privilege he refused to answer, but it was decided by Vice-Chancellor Kindersley that he had no privilege, but was bound to tell all he knew, and this decision put him to an expense of £30 for costs. In cases of a criminal kind, the same point has several times arisen, and it has even happened that the reply made by the accused to a doctor's professional question has been the sole evidence on which a conviction could be based. It will be perceived, therefore, that any statements which are made to physicians or surgeons while attending persons in a private capacity, although they are not to be volunteered in evidence, must be given in answer to questions whatever consequences may ensue.

---

Owing to the rapid development of an outbreak of measles in Liverpool, the Education Authorities, acting on the advice of the Medical Officer of Health, on March 15 issued an order for the immediate closing of all infant schools in the city. This order affects 158 schools, at which 39,000 children are in attendance; and it is to operate in the first instance for a fortnight, during which time the effect will be watched both by the medical staff and the education authorities. The outbreak is not confined to one district, but is spread all over the city. Last week 16 deaths from measles and 226 new cases were reported.

